# Twin Study Design

**Published:** 1995

**Authors:** Carol A. Prescott, Kenneth S. Kendler

**Affiliations:** Carol A. Prescott, Ph.D., is co-director of the Stress & Coping Twin Project and assistant professor of the Psychiatric Genetics Research Program, Department of Psychiatry, Medical College of Virginia/Virginia Commonwealth University, Richmond, Virginia. Kenneth S. Kendler, M.D., is director of the Psychiatric Genetics Research Program and is Rachel Brown Banks Distinguished Professor in the departments of psychiatry and human genetics, Medical College of Virginia/Virginia Commonwealth University, Richmond, Virginia

**Keywords:** twin study, AOD dependence, hereditary factors, environmental factors, applied research, gender differences

## Abstract

By studying human pairs of twins, researchers can learn the relative contributions of genetic and environmental factors to the development of alcoholism. Identical (i.e., monozygotic, or MZ) twins share 100 percent of their genes, whereas fraternal (i.e., dizygotic, or DZ) twins generally share only 50 percent of their genes. Using certain techniques and theoretical models, researchers can compare the two types of twin pairs for how often alcoholism occurs in both members of a twin pair. If alcoholism occurs more often in both members of MZ twins, genetic factors are implicated in the origin of the disorder. Twin research also has been applied to studies of differences between men and women in their genetic contribution to alcoholism.

Humans are biologically similar, sharing almost all of their genetic material. Many questions relevant to alcoholism,^1^ however, concern how people differ. Why do some people abuse alcohol? Why do some heavy drinkers, but not others, become physiologically addicted to alcohol? Twins are a unique resource for identifying the genetic and environmental sources of these differences. The study of the causes of differences (i.e., variation) among people is a major goal of human behavioral genetic research, of which twin studies form a subset.

Twin studies have been used to address important questions about the causes of alcoholism, including the following:

- How important to the development and course of alcoholism are genetic and environmental influences?
- Do genetic influences differ in importance for different groups of people (e.g., males and females or different age groups)?

Based on partial answers from these investigations, new twin studies are seeking answers to even more specific questions, such as the following:

- Do the same sets of genes (or the same environments) produce the same effects in males and females?
- What environmental characteristics are most hazardous for people at high genetic risk for developing alcoholism?
- What environmental characteristics *protect* against alcoholism among people who have a strong family history of the disorder?
- To what degree is the frequently seen overlap between alcoholism and other disorders (such as depression and anxiety) a result of the same sets of genes influencing both disorders?

These questions have important implications for understanding the causes of alcoholism and ultimately providing knowledge to help design treatment and prevention strategies for people at high risk of developing the disorder. This article describes how twin studies are designed, offers examples of research questions for which twin studies are useful, and reviews their limitations.

## The Twin Study Design

### The Value of Twins

Because of their unique genetic status, twins play a valuable role in teasing apart genetic and environmental influences on people’s development of alcoholism. Genes are segments of DNA that form the blueprints for the development of the human body. Genetic expression that influences behavior is only partly preprogrammed; the biological processes regulated by genes are modified in complex ways by experiences with the environment. Thus, neither genes nor experiences operate independently of one another. In this sense, the often-cited dichotomy of “genes versus environment” is false; both are required for the development and expression of human characteristics. Knowledge of how genetic and environmental factors act both separately and together to influence the development of alcoholism can inform strategies (e.g., biological, individual, or societal) for intervention and prevention.

When researchers study genetic influences on behavioral variation, they focus on the small percentage of genes that differ among people. Fraternal, or dizygotic (DZ), twin pairs, like ordinary siblings, have, on average, 50 percent of these genes in common, whereas identical, or monozygotic (MZ), twin pairs have 100 percent of these genes in common. By comparing MZ with DZ twin pairs, researchers can use this difference in the twins’ degrees of genetic resemblance to estimate the relative importance of genetic and environmental influences on the development and course of disorders such as alcoholism.

### Liability Models

**Figure 1:**
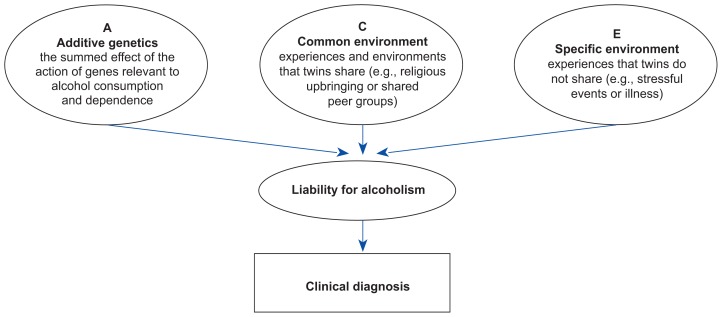
Components influencing a twin’s risk (i.e., liability) for developing alcoholism. Three theoretical components—additive genetic sources (A); common environmental sources (C), which twins in a pair share; and individual, specific environmental sources (E)—combine to create a person’s liability for developing alcoholism. In this example, liability is theoretical and can only be inferred from the observed clinical diagnosis.

When searching for genetic and environmental influences on alcoholism, researchers can only observe a characteristic in a subject, such as the presence or absence of the clinical diagnosis of alcoholism. Researchers, however, are actually trying to estimate the contribution of genetic and environmental effects to the subject’s *liability* for alcoholism (the theoretical components contributing to the risk for alcoholism are portrayed in figure 1). Liability may be thought of as the outcome of genetic and environmental risk factors that together produce a person’s total risk for developing alcoholism (Falconer 1965). Liability is an unobserved, or latent, characteristic that varies in the population.

The three components of liability usually identified in twin research are as follows:

- Additive genetic liability, or the summed effect of many genes relevent to alcohol consumption and dependence
- Common environmental effects that twins share (e.g., parental influence, intrauterine environment, and sociocultural factors)
- Environmental effects that twins do not share (e.g., experiences such as marital problems or job loss). (For further discussion, see sidebar, pp. 204–205.)

**Determining Liability**

To estimate the relative importance of genetic and environmental sources of a characteristic, such as a person’s liability for developing alcoholism, researchers use mathematical equations in twin studies. In the most common model, liability for alcoholism is attributed to three unobserved, or latent, sources: additive genetic (A); common environmental (C); and individual, specific environmental (E). Each type is described below.

***Additive Genetic Sources***. Humans have 46 chromosomes,^1^ a number that includes 22 pairs plus the sex chromosomes, XX or XY. The genes in a pair may be similar but not identical; a gene for a particular trait on one chromosome may vary slightly from the corresponding gene on the paired chromosome. Such gene variants are called *alleles*.

The effects of alleles on observed characteristics combine in three major ways. Nonadditive genetic mechanisms include *dominance* (a term that describes any interaction between paired alleles other than a simple summing of their effects) and *epistasis* (wherein alleles at different sites alter the effects of each other). However, the most common mechanism of allelic action for complex behavioral traits is believed to be through *additive genetic action*. In the case of alcoholism, this would mean that all alleles relevant for the development of alcoholism combine and are not suppressed or magnified by alleles on the paired chromosome or any other location. The A component estimated in twin studies represents the total additive genetic action relevant to a person’s risk for developing alcoholism. Most twin models assume that dominance and epistasis are negligible. For example, genes that predispose a person to have a higher risk for alcoholism (e.g., by increasing alcohol’s euphoric effects) might combine with other genes that reduce risk (e.g., by producing a flushing response) to produce a moderate level of risk. Identical, or monozygotic (MZ), twins have 100 percent of their genes—including those that influence risk for alcoholism—in common, whereas fraternal, or dizygotic (DZ), twins share (on average) only 50 percent of the genes that vary in the population (see figure).

***Common Environmental Sources***. Common environmental influences on problem alcohol use might include parental drinking habits and teachings about alcohol, shared peer groups, and sociocultural influences. For twins (but not other siblings), these components also include the prenatal environment. In studies, researchers assume these factors correlate perfectly for twins or siblings reared together, shown by a correlation of 1.0 in the figure for the C components.

***Individual, Specific Environmental Sources***. These include any experiences or environmental influences not shared by siblings, such as unshared peers and stressful life events (e.g., job loss, marital problems, or physical illness). The estimate of this term usually includes random measurement error (such as that from misdiagnosis), which cannot be separated from E in studies in which twins are measured only once. Because the members of a twin pair do not share specific environmental sources, no correlation exists between the E components in the figure shown.

***Liability Models***

Under the usual liability model, the three components described above are assumed to combine. The liability (*L*) for an individual (*i*) can be expressed as: *L_i_* = *a*(*A_i_*) + *c*(*C_i_*) + *e*(*E_i_*). The uppercase letters A, C, and E represent theoretical “scores” on each of the three components; these scores vary across people. The lowercase letters a, c, and e designate the amount each component contributes to the outcome, and these are constant for everyone in a group.

The similarity, or correlation, in liability for two people is estimated as the sum of the components that they have in common. Because members of MZ twin pairs are identical for both their A and C components, their correlation is estimated as *a^2^* + *c^2^*. Members of DZ twin pairs share one-half their genes and all their common environment; therefore, the correlation for them is 0.5(*a^2^*) + *c^2^*. With just these two correlations, the values of a, c, and e can be determined using algebraic rules.

**Figure:**
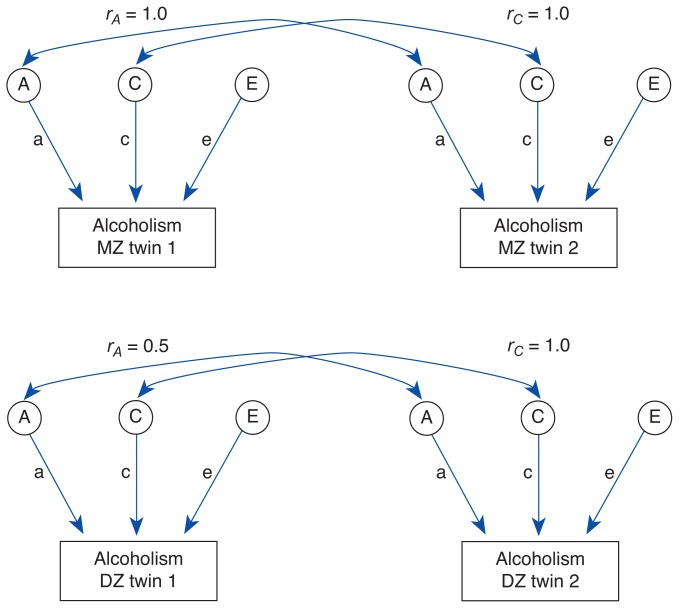
Model demonstrating how three factors determine twins’ resemblance for liability for alcoholism. The genetic makeup (A) of identical (monozygotic [MZ]) twin pairs (upper section) is correlated perfectly (represented as *r_A_* = 1.0), whereas the genetic makeup of fraternal (dizygotic [DZ]) pairs (lower section) is correlated to only one-half the degree of MZ pairs (*r_A_* = 0.5) for their genetic components. Because each set of twins was raised in the same environment, both types are correlated perfectly (*r_C_* = 1.0) for common environmental components (C) and uncorrelated for individual, specific environmental components (E). Thus, resemblance for liability between DZ twins results less from genetic factors than does resemblance between MZ twins.

For a simplified illustration of these formulas at work, imagine that a study finds that MZ twin pairs correlate at +0.7 for alcoholism or not, whereas DZ pairs correlate at +0.4 for this characteristic. This pattern would occur if MZ pair members were much more likely than DZ pair members to both be alcoholic or both be nonalcoholic. Inserting these numbers into the equations above [0.7 = (*a^2^* + *c^2^*); 0.4 = (0.5(*a^2^*) + *c^2^*)] results in estimates of *a^2^* = 0.6, *c^2^* = 0.1, and *e^2^* = 0.3. This means that 60 percent of the differences among people in the tendency to develop alcoholism would be attributable to differences in their genotypes, 10 percent would be attributable to environmental factors shared with siblings, and 30 percent would be attributable to unshared environmental effects.

Note that the scores A, C, and E are not estimated; standard twin studies provide estimates of a, c, and e, and the overall contribution of these components for a whole population, but usually do not provide estimates of the genetic and environmental levels of risk for individual persons. Researchers in the field of behavior genetics, however, have begun to include measured aspects of the environment in their models and, as molecular genetic technology improves, will be able to include measures of specific genetic regions (see the article on quantitative trait loci by Grisel and Crabbe, pp. 220–227).

—*Carol A. Prescott and Kenneth S. Kendler*

1For a definition of this and other technical terms used in this sidebar, see central glossary, pp. 182–183.

Researchers assume that the three components listed above combine to produce total liability. Only people who exceed a specific level, or threshold, of liability will manifest the clinical aspects of the disorder. Thus, people with low genetic liability require higher levels of environmental risk before they are likely to develop alcoholism, whereas those with high genetic liability may develop alcoholism in response to lower levels of stressful events.

## Estimates From Twin Studies

At conception, MZ twins are genetically identical. Although mutations in some genes are likely to occur, and adult MZ twins may differ in which genes currently are active (because of unshared environmental influences, such as disease or nutrition), these differences are believed to be small. MZ twins are assumed in studies to be perfectly correlated for genes relevant to the development of alcoholism. Because first-degree relatives, including DZ twins, have, on average, one-half their genes in common, DZ twins have only one-half the degree of genetic correlation seen between MZ twins. Thus, if members of MZ twin pairs are much more alike for liability for alcoholism than are DZ twins, genetic influences can be implicated as a major contributor to liability. Conversely, if MZ and DZ twin pairs are equally similar for alcoholism liability, then any similarity is attributed to shared environmental factors. Researchers use equations based on the MZ–DZ comparison to estimate the proportions of liability resulting from the genetic and environmental components (for further discussion, see sidebar, pp. 204–205).

## Applying the Twin Design

The twin design frequently has been applied to studying differences in the relative importance of genetic and environmental influences among different groups, such as males and females or different age sets (i.e., cohorts). For example, the estimates of genetic influence from studies of male twin pairs could be compared with the estimates resulting from female twin pair studies to identify differences between the groups. Other variations on traditional twin study designs could address important questions about differences in the mechanisms of genetic influence (discussed below).

### Sex Differences

Although the risk for alcoholism may vary somewhat in different demographic groups (e.g., by age, ethnicity, or socioeconomic level), the difference between sexes in prevalence of alcoholism is particularly dramatic. Because the genetic differences between males and females are larger than between any other groups, comparing the sexes can provide more specific information about genetic mechanisms of risk for alcoholism.

#### Sources of Liability

Studying male and female same-sex pairs allows the calculation of separate estimates of genetic and environmental effects for each sex. Although the data can show if the sexes differ in the relative importance of genetic and environmental sources of liability for alcoholism, the data cannot determine whether these genetic and environmental influences are the same in both sexes. Researchers attempt to solve this question by studying opposite-sex twin pairs (which are never MZ pairs).

By using opposite-sex twin pairs, sex differences in genetic and common environmental sources of liability sometimes can be estimated. For example, if DZ opposite-sex twin pairs are less similar in their alcoholism liability than DZ same-sex pairs, this finding would be evidence that the two sexes differ either in the genes or environments (or both) relevant for the development of alcoholism. For statistical reasons, the twin design cannot always reveal the degree to which these differences result from genetic or environmental distinctions between the sexes. Evidence that such differences exist, however, provides an important starting point for researchers studying sex differences in alcoholism. Thus far, the available opposite-sex twin data are limited (for review, see McGue 1994), but two large twin studies that include male-female pairs are underway in Australia (A. Heath and colleagues, ongoing research) and in Virginia (K. Kendler and colleagues, ongoing research).

#### Transmission of Liability

Other applications of the twin design address the following questions about sex differences in the mechanisms responsible for transmitting liability for alcoholism between generations:

- Do mothers and fathers differ in the amount of genetic liability they pass to their children?
- Once genetic influences are accounted for, do parents contribute any further environmental risk by their behavior?
- Do mothers and fathers differ in the magnitude or mechanism of environmental liability they pass to their children?
- Does an alcoholic parent contribute different levels of risk to sons versus daughters?
- Does the importance of parent-offspring transmission differ in different age cohorts?

These questions require the use of twin-family designs, in which both twins and their parents are studied. Results from one such study of female twins and their parents indicated that mothers and fathers were equally likely to transmit liability for alcoholism to their daughters (Kendler et al. 1994).

## Heterogeneity of Expression

Another major application of the twin model is in the study of the different ways the same genetic liability can be expressed, often called *phenotypic heterogeneity*. For example, the same genetic liability may produce different clinical presentations (or *phenotypes*) in the sexes: A woman may become depressed, but a man may become alcohol dependent. In another example, genetic liability may be expressed as alcoholism in one generation and drug dependence in another. Studying opposite-sex twin pairs or twins from different age cohorts can address these important issues regarding the origins of alcoholism and its overlap with other disorders.

Another type of difference in expression of liability is called *etiologic heterogeneity*—when the same clinical disorder (e.g., alcoholism) occurs through multiple pathways. For example, women with alcoholism are at higher risk for anxiety and depression than are alcoholic men, whereas alcoholic men are more likely than women to have antisocial characteristics (Helzer and Pryzbeck 1988). These data, in combination with results from family studies, have led researchers to propose that alcoholism in men is primary (i.e., it occurs first) and is often associated with sociopathy, whereas alcoholism in women is more commonly secondary to other emotional disorders (see, for example, Cloninger and Reich 1983; McGue et al. 1992).

By examining the types of disorders present in the co-twins of alcoholics (e.g., antisocial personality versus depression), researchers also can better understand the clusters of symptoms and subtypes that constitute alcoholism. Furthermore, these studies can show whether the basis for the overlap is genetic, environmental, or both. For example, Kendler and colleagues (1994) found that the co-occurrence of alcohol dependence and depression among female twins was attributable primarily to shared genetic, not shared environmental, risk factors.

Genetic liability for alcoholism also may interact and combine with genetically influenced characteristics other than sex (e.g., personality type) or with environmental influences (e.g., choice of peer group or religious or cultural prohibitions), resulting in varying outcomes for different people. More sophisticated behavioral genetic models are being developed that can address how these factors may mediate the development of alcoholism.

## Limitations of Twin Studies

As with any research design, results from twin studies are generalizable only to the extent that twins are representative of the entire population. Evidence indicates that the prevalence of psychiatric symptoms among twins does not differ from the prevalence in the general population (Kendler et al. in press). People willing to participate in research studies, however, may differ from random samples (e.g., by the severity of their disorder or by their socioeconomic level), leading to potential biases in the study population.

Some studies obtain alcoholic twin subjects from treatment settings, whereas other studies assess twins from the general population. Because treatment-based studies typically identify only a minority of the affected cases in the general population, researchers must determine whether these cases differ in severity or symptomatology from those not included in the research. A particularly important consideration for twin research is whether twins in studied pairs are more likely to be similar than pairs in the general population. For example, twin pairs in which both twins are alcoholic may be more likely to participate in treatment or attract the attention of researchers (and thus be included in studies) than pairs in which only one member is alcoholic, thereby leading to biases in estimates of twin similarity (see, for example, Prescott and Kendler 1995). Similarly, studies based on twins in the general population are subject to volunteerism biases. Females and identical twins are more likely to participate in research than are males or fraternal twins, and people with alcohol-related problems are less likely to be willing to participate in research; they also can be difficult to locate. The effects of such biases can be examined using statistics, but only if the biases are identified and measured.

Other limitations of the twin method arise from the assumptions required to estimate genetic and environmental effects. The standard twin design assumes the absence of *assortative mating* (i.e., the tendency for people to choose mates who are similar to themselves with respect to the characteristic being studied). Alcohol consumption, however, is an integral aspect of social activity for many people, and spouses tend to resemble each other regarding alcohol use and misuse (see, for example, Jacob and Bremer 1986). When assortative mating for alcoholism occurs, offspring who are DZ twins share more than 50 percent of alcoholism-relevant genes, leading to an underestimation in twin studies of the magnitude of genetic influence. It is possible, however, to correct the results from twin studies by using estimates from other studies of spousal similarity with respect to alcoholism.

Another crucial assumption that can limit the accuracy of twin studies is the *equal-environment assumption*. This is the assumption that MZ and DZ twin pairs are equally similar in their shared environments. To the extent that this is not true, the importance of a shared environment will be underestimated and genetic influence overestimated. For example, if peers are an important influence on drinking behavior, and MZ twins are more likely to share peers with their twin than are DZ twins, the greater MZ similarity will be attributable to environmental as well as genetic causes but will be ascribed solely to genetic influences.

Attempts to address the equal-environment assumption with respect to alcohol use and misuse have yielded mixed results (Kendler et al. 1992; Lykken et al. 1990; Rose et al. 1990), with some studies finding that twins who spend more time together (often MZ pairs) are more alike in their drinking habits. Even after statistically controlling for the effects of greater environmental similarity among MZ twin pairs, however, substantial genetic influences on alcohol intake remain (see, for example, Kendler et al. 1992).

A further limitation of the twin study design is that the separation of liability into distinct genetic and environmental components may be invalid if sizable gene-environment *correlations* or *interactions* exist. A gene-environment *correlation* occurs if persons with high genetic liability for alcoholism are more likely to experience alcoholism-promoting environments (this may occur among children of alcoholics, who may obtain both genetically and environmentally transmitted risk for alcoholism). Gene-environment *interactions* occur when persons with high genetic risk are particularly sensitive to the effects of alcoholism-promoting environments. Twin data can be applied to these issues if a range of environmental conditions is available for study. Data from studies of adopted children also are useful for testing for gene-environment interactions, but these studies have other methodological limitations. (For further discussion of adoption studies, see the article by Cadoret, pp. 195–200.)

## Summary

Twin studies can be a powerful tool for addressing important questions about the development and course of alcoholism, including the role of genetic and environmental factors, as well as sex differences in the mechanisms and the magnitude of genetic and environmental influences. The results from opposite-sex twin studies, along with those from adoption and twin-family studies, promise to improve our understanding of the roles of genetic factors and of experiences and their interactions in the causes of alcoholism.

## Footnotes

1 The term “alcoholism” is used in this article to encompass all levels of problem alcohol use and does not refer to a particular diagnostic system. The term “alcohol dependence” is used when referring to research that used this diagnosis as defined by the *Diagnostic and Statistical Manual of Mental Disorders, Third Edition, Revised* of the American Psychiatric Association (1987).
